# Can Individual Attitudes toward Aging Predict Subsequent Physical Disabilities in Older Taiwanese Individuals? A Four-Year Retrospective Cohort Study

**DOI:** 10.3390/ijerph18010098

**Published:** 2020-12-25

**Authors:** Chien-Yao Sun, Chun-Yin Yeh, Yan Zhao, Ching-Ju Chiu

**Affiliations:** 1 Division of Geriatrics and Gerontology, Department of Internal Medicine, National Cheng Kung University Hospital, College of Medicine, National Cheng Kung University, Tainan 70101, Taiwan; chienyaosun@gmail.com; 2 Department of Computer Science and Information Engineering, College of Electrical Engineering and Computer Science, National Cheng Kung University, Tainan 70101, Taiwan; annayayadunk@gmail.com; 3 WHO Collaborating Centre for Community Health Services, School of Nursing, The Hong Kong Polytechnic University, Hong Kong 999077, China; yan-ivy.zhao@polyu.edu.hk; 4 Institute of Gerontology, College of Medicine, National Cheng Kung University, Tainan 70101, Taiwan

**Keywords:** attitudes toward aging, aging perceptions, physical function, older adults

## Abstract

Individual attitudes toward aging have been regarded as a modifiable risk for physical disability. However, longitudinal cohort studies have not been carried out in countries in Asia. In the present study, we aimed to explore the association between individual attitudes toward aging and subsequent physical disabilities using a nationwide representative cohort, the Taiwan Longitudinal Study on Aging (TLSA), over a 4-year follow-up period. In 2003, a baseline survey for 10-item attitudes toward aging scale consisting of widely different domains across financial relationships with children, grandparenting, living arrangements, and remarriage was conducted. Later, physical disabilities, including mobility and activities of daily living (ADL) limitations, were evaluated in 2007. A total of 1635 participants aged 57 and over were analyzed. Older age, self-rated poor health, and those suffering from pain were found to be more likely to have higher risk of physical disabilities. The older adults who expressed a willingness to receive financial support from their adult children were reported to have a lower risk of mobility limitations (adjusted odds ratio (aOR): 0.67, 95% confidence interval (CI): 0.50–0.90), while those who did not want to assist with child care as grandparents had a higher risk of ADL difficulties (aOR: 2.46, 95% CI: 1.31–4.60). Our work shed light on the importance of individual attitudes toward aging in predicting long-term physical disabilities and illuminated the intimate role of grandparents, both financial and participatory, in Chinese families. In the future, culturally adapted attitudes toward aging scale should be developed to identify older Chinese adults at risk of physical disabilities.

## 1. Introduction

Taiwan is one of the most rapidly aging countries. A significant proportion (7.5%–11.5%) of older people in Taiwan are reported to have physical disabilities, including mobility limitations or deficits in the basic activities of daily living (ADL) [1]. It is well-known that physical disabilities can lead to poor health outcomes and may even increase mortality rates in older people [2,3]. Several potential modifiable physical and psychological factors have been investigated to determine their ability to prevent physical disabilities [4,5]. More recently, attitudes toward aging, which refers to personal expectations or perceptions of one’s own aging process [6,7], have been linked to health status, including cognitive performance, physical capacity, and medical utilization [8,9].

Attitudes toward aging have been gradually recognized as a psychological factor exerting influence independently on the physical functions of older people [7,8,10,11]. Previous work has postulated the plausible biopsychosocial mechanism linking mind to body [12,13]. However, different operationalized measures of attitudes toward aging have been established, such as the positivity or negativity associated with self-perceptions of aging [8]. In addition, attitudes toward aging in older people may be affected by a complex interrelationship of social participation, cultural background, historical context, and the meaning of one’s life [9,14]. Notably, attitudes toward aging may alter at different time points over one’s life span [15]. Previous studies have been conducted in Western countries examining the impact of attitudes toward aging on physical indices [7,10,11,16,17]. Moser et al. found that self-perception of aging in Switzerland based on a five-item scale could be used to determine vulnerability to adverse ADL difficulties after adjusting for socio-economic characteristics, chronic medical conditions, living arrangements, and depressive symptoms in 1422 participants aged 65–70 years [7]. However, the baseline physical status was not taken into account to determine the relationship between self-perception of aging and subsequent physical disabilities [7].

A cohort study examining the associations between attitude toward aging and physical disabilities has not been carried out in Asian countries. In the present study, we aimed to explore the longitudinal association between individual attitudes toward aging and physical disabilities using a nationwide representative cohort over a four-year follow-up period. We hypothesized that the baseline attitude toward aging may be associated with subsequent physical disabilities in older adults in Taiwan independent of significant confounders. 

## 2. Materials and Methods

### 2.1. Study Design and Data Source

This was a retrospective study drawing data from the Taiwan Longitudinal Study on Ageing (TLSA). The TLSA was established in 1989 and was aimed toward coping with the impact of population aging in Taiwan. The nationwide representative survey was initiated by the Taiwan Health Promotion Administration in collaboration with the University of Michigan, which conducted the longitudinal panel study (Health and Retirement Study, HRS) in America. The sampling for the longitudinal survey involved the use of a three-stage sampling (1989, 1996, 2003), seven-wave survey (1989, 1993, 1996, 1999, 2003, 2007, and 2011), and stratified (sex, age, districts) systematic random sampling framework to obtain a nationally representative sample in Taiwan. The first interview was conducted in 1989 with people aged 60 years and older enrolled. The follow-up cohort sampling in 1996 and 2003 recruited participants aged 50 years and older. It was conducted with face-to-face interviews using structured questionnaires confirmed by an expert panel consisting of background information, household information, work history, and health status, which details have been described elsewhere [18]. A high response rate (80–90%) was noted. 

The data used in this research was the survey for attitude toward aging in 2003, containing the second-stage cohort enrolled since 1996, and later a complete evaluation for physical status in 2003 and 2007. Overall, a total of 1635 participants were enrolled for final analysis. All the participants provided informed consent with preserved anonymity. The study followed the ethical guidelines in the declaration of Helsinki and was approved by the Human Research Ethics Committee at National Cheng Kung University (B-ER-104-077).

### 2.2. Definition and Description of Variables

#### 2.2.1. Attitudes toward Aging

In this study, attitudes toward aging were the primary predictor variables. In the TLSA, attitudes toward aging comprised 10 items involving multiple culturally adapted dimensions. (1) When parents are old, children should give them money to spend on necessities (children paying expenses); (2) if the older parents keep some savings or their property, their children will treat them with more respect (keeping property for respect); (3) if their children are in need, parents should help them take care of their children (grandchildren) (assistance with taking care of grandchildren); (4) parents should try their best not to live with their married children (avoid living with married children); (5) older parents should be as financially independent as possible instead of relying on their children (financial self-reliance); (6) today’s young people are less respectful of older people than in the past (young people today have less respect for older people); (7) the government is already providing good care for older people (the government takes good care of older people); (8) when parents get old, their children should live with them (living with children); (9) if an older adult’s spouse passes away, are you in favor of his remarriage (widower’s remarriage)? (10) If an older woman’s spouse passes away, are you in favor of her remarriage (widow’s remarriage)?

These questionnaires were scored from 1 to 5: 1 for strongly agree and 5 for strongly disagree. Participants who reported “strongly agree” or “agree” were combined into the “agree” category, and the rest were categorized as “disagree.” In this study, the answers expressing agreement were treated as the reference group. The 10 dimensions shape individual attitudes toward aging differently and at varying magnitudes. Hence, a factor is not perfectly correlated with any dimension of attitude toward aging. 

#### 2.2.2. Other Key Covariates

Baseline status in 2003, including socio-demographic characteristics, several self-reported chronic conditions, depression, and physical status served as the key confounding variables in this study [7].

The socio-demographic characteristics comprised gender, educational level, marital status, work status, income, and living alone. The self-reported chronic conditions included self-described health, number of health problems, pain, and hospitalization within the previous year.

Depression status was assessed using 10 items from the Center for Epidemiologic Studies Depression Scale (CES-D Scale), which was adapted from the original scale developed in 1997 [19] with good reliability and criterion validity [20]. The total scores of the CES-D scale range from 0 to 30, with a higher score indicating a higher level of depression (0: no depression, 1–9: low-level depressive symptoms, 10–30: high-level depressive symptoms). 

#### 2.2.3. Physical Disability

Two outcomes related to physical disability were measured as the primary outcomes of interest.

First, mobility was assessed according to nine items, including standing for about 15 min, standing for 2 h, squatting, hands on the head, taking or reversing things with fingers, picking up or carrying things weighing 20 pounds, short distance running (20–30 m), completing 200–300 m of walking, and being able to walk up to the second floor or third floor of a building [21]. Each item was divided into no difficulty (0), some difficulty (1), very difficult (2), or completely disabled (3), and those who answered 1–3 to any of the questions were classified as having “mobility limitations.” Second, the basic activities of daily living (ADL) were evaluated using six items including taking a bath, taking off and putting on clothing, eating, getting up/standing/sitting in a chair, walking indoors, and toileting [22]. ADL limitations were similar to mobility limitations.

### 2.3. Data Analysis

The SAS 9.4 software (SAS Institute Inc., Cary, NC, USA) package was used. All continuous variables were expressed as mean ± SD, and the categorical variables were summarized by numbers and percentages. The multivariable logistic regression model was applied to examine the relationship between attitudes toward aging and physical disabilities. In the multivariable logistic regression analyses, we adjusted all the following potential confounders, socio-demographic characteristics, self-reported chronic conditions, and depression status based on clinical observation and theoretical hypothesis. Sensitivity analysis was conducted with all indicated variables to compete, and those items with alpha value less than 0.20 in univariable regression were allowed to enter for a further stepwise logistic regression model. All statistical tests were two-tailed, and statistical significance was set at *p*-value < 0.05.

## 3. Results

### 3.1. Participant Characteristics

Table 1 shows the baseline characteristics, self-reported chronic conditions, depression status, physical disabilities, and responses to the attitude toward aging scale in 2003. Among the 1635 participants, 847 were men (51.80%), with a low education level (illiterate or elementary school: 84.28%), and 1307(79.95 %) were married. Over 70% of the participants were unemployed, and less than 10% lived alone. Regarding the self-reported chronic conditions, less than half of the participants rated themselves in good health. More than one disease was identified in 80.31% of the study sample, and tolerable pain was noted in 47.95%. Just over twelve percent (12.35%) of the participants had been hospitalized within the past year, and depressive symptoms (low to high levels) were quite common (95.54%). ADL limitations were noted in 71 (2.72%) of the participants, and one-third had mobility limitations. Regarding attitude toward aging, most participants agreed with “children providing expenses” (89.05%), followed by “assistance with taking care of grandchildren” (88.38%), and “keeping property for respect” (84.89%). In contrast, the participants mostly disagreed with intimate relationships after becoming a widower (78.17%) or widow (83.36%).

### 3.2. Associations between Individual Attitudes toward Aging and Subsequent Physical Disability

During the four-year follow-up period, the study population experienced a higher percentage of physical disabilities (mobility limitations: 66.51%, ADL limitations: 11.34%, respectively). Table 2 shows the results of the logistic regressions, providing the associations between attitudes toward aging and subsequent physical disabilities in 2007 while controlling for socio-demographic characteristics, self-rated status, and baseline physical function. Those who were older (adjusted odds ratio [aOR] = 1.09, *p* < 0.0001) and female (aOR = 1.96, *p* < 0.0001) had a higher risk of mobility limitations after controlling for the baseline mobility status. Those with self-rated poor health (aOR = 2.04, *p* < 0.0001), more than one disease (aOR = 1.35, *p* = 0.05), and tolerable pain (aOR = 1.76, *p* < 0.0001) had a significantly higher risk of mobility limitations. Notably, those who disagreed with the statement that “older parents should be as financially independent as possible instead of relying on their children” had 33% lower risk of mobility limitations than those who did not (aOR = 0.67, *p* = 0.01).

Those who were older, lacked an income, had self-rated poor health, and were experiencing pain were reported to have a higher risk of ADL limitations. In addition, participants who disagreed with providing assistance with taking care of their grandchildren had a 2.46 times higher risk of ADL limitations, after controlling for baseline ADL status. The association between attitude items and physical disabilities remained similar after excluding those with neutral response. Also, a sensitivity analysis was conducted with variables allowed to enter multivariable analysis if the alpha value was less than 0.20, which reveals similar findings, with the effect of grandparenting remaining robust and financial self-reliance modestly weakened.

## 4. Discussion

It was found in the present study that individual attitudes toward aging can predict physical disabilities in older adults in Taiwan over a four-year follow-up period. To the best of our knowledge, this is the first study using population-based representative data to explore the association between attitudes toward aging and long-term follow-up health outcomes in Asian countries. Older adults who agree with the concept of financial self-reliance and disagree with assisting with grandparenting were shown to have a significantly higher risk of subsequent physical disabilities independent of demographics and baseline risk factors. 

Our study findings indicated that the older subjects who disagree that “the elderly should be as financially independent as possible instead of relying on their children’s resources” had a 33% lower risk of mobility difficulties, which is somewhat different from the findings of previous research [23]. Miller et al. conducted a systematic review to investigate the risk factors related to health outcomes among older patients in the United States. Their results showed that older people with low incomes are more likely to suffer declines in physical functions [23]. Nonetheless, more environmental resources may increase the complexity of the external stresses that older people have to cope with. Previous studies have assumed that financial support from adult children may represent filial piety in offspring, whereas older Chinese participants with high expectations of filial piety were reported to have poor physical health [23]. The relationship between disagreement with financial self-reliance and better physical health is not fully understood, although the role adjustments required of older people with the family may play a moderating role. Based on the old adage, “It is more blessed to give than to receive,” it might be beneficial to engage older adults in family affairs while providing them with financial support. In future studies, role adjustments and engagement in family affairs for older people might need to be taken into account to examine the relationship between older people’s expectations of financial self-reliance and subsequent physical disability.

In this study, the risk estimate of ADL limitations in older participants who disagreed that they should assist with childcare in their role as a grandparent was 2.46 times higher than that in their counterparts. The results were similar to a work conducted by Ku et al., where it was found that older adults being caregivers to their grandchildren were more likely to experience health advantages in the areas of both physical and mental health [24]. Previous studies regarding attitudes toward aging rarely address the role of intimate relationships, including grandparenting, among older adults and family members in Western countries (Switzerland, the United Kingdom, the United States, Australia) [7,10,25,26]. In contrast to Western culture, older adults in Taiwan believe that the family is the basic unit of society, wherein the individual and family are inseparable [27]. A survey of the situation of senior citizens in Taiwan showed that most of the respondents (89.9%) lived with their adult children and grandchildren, which was additionally reported to be the best living situation for older adults [28]. The intimate relationship made it possible for these older adults to exercise their responsibilities, which resulted in a better health status and significant improvements in life satisfaction among the older people in Taiwanese society. A model of successful aging developed by Rowe and Kahn [29] in a longitudinal follow-up cohort of 1,189 healthy elderly individuals found that maintaining intimate relationships with others and continuously taking part in meaningful daily activities is one of the important components to achieving successful aging. In the older adults in Taiwan considered in the present study, the results emphasized grandparenting as one of the attitudes toward aging in Chinese culture that may play a protective role in maintaining better physical functioning.

Considerable research has addressed the importance of attitudes toward aging on physical function [7,8]. Similarly, our results note that individual attitudes toward aging was associated with physical health independent of chronic illness and baseline physical status in an aged Asian population. The biopsychosocial mechanism is not fully understood despite previous exploring research. Levy et al. found that individual attitudes toward aging may be internalized unconsciously and influence health through multiple pathways [30]. A longitudinal cohort of middle-age or older adults has demonstrated the correlation of attitude toward aging and increased levels of stress-related inflammatory biomarker, and even shortened survival [13]. A stress-related pathological pathway that may link individual attitude toward aging to physical function is suggested. Alternatively, attitude toward aging may influence health consequences through non-stress pathways, such as decreased motivation and sedentary behavior [12]. There is substantial research highlighting the unfavorable association between sedentary behavior and health outcomes through various biological mechanisms, including insulin resistance via homeostatic model assessment, blood lipid (high or low density lipoproteins, lipoprotein(a)), and anthropometric (body mass index, fat mass, waist circumference) [31]. Taken together, these results suggest that attitude toward aging affects physical health through stress and non-stress-related pathophysiological pathways linking mind to body. Future research is warranted to explore the potentially distinct pathways by which attitude toward aging affects physical health. Moreover, attitude toward aging appears likely to be a suitable intervention target to promote physical health among older adults. 

Our study has several strengths. First, this work has a longitudinal design with a lengthy follow-up Second, these data were derived from a national-wide representative cohort of a community-dwelling older Asian population with a large sample size. Third, the present study shed light on the roles of individual attitude toward aging in long-term physical health in non-Western culture. However, this study also has a few limitations and should be interpreted cautiously. First, although the findings emphasize the benefits of grandparenting, the possible downsides including complications derived from the caregiving process were not taken into consideration. Second, the scope of our study was confined to financial reliance from family support among the older adults but did not include a larger context, like social support. Third, despite our attempt to include all possible confounders, several residual unmeasured confounding variables were not enrolled in the adjustment process, such as frailty status, nutrition, biochemistry values, and disease severity. Fourth, self-reported questionnaires might interfere with the risk estimation for recall bias, though the misclassification tended to be non-differential. Otherwise, despite a high rate of mobility and ADL limitation events, possible inflation of type 2 errors cannot be ruled out with only 2 of the 10 attitude items being outcome predictors. Finally, this data is 12 years old and the validity of the attitude toward aging screening tool in TLSA remains uncertain. This significant issue is worth further investigation and will be included in our future work. An additional longitudinal cohort study is warranted to validate our study findings. 

## 5. Conclusions

In summary, the present study highlighted the importance of individual attitudes toward aging in predicting long-term physical disabilities independent from socio-economic characteristics, chronic disease, depressive symptoms, and baseline physical status. Unlike the Western population, older Asian adults who were provided financial support from adult children and assisted in grandparenting tended to retain physical functioning. These findings also shed light on intimate relationships, both financial and role participation, in Chinese families. Aging individuals may benefit from caring for children from a physical perspective more than the financial support they may be receiving. These findings may facilitate preventive strategies against functional decline in medical efforts for older people. Our study provided a rationale for future development of a culture-adapted attitudes toward aging scale and longitudinal research to prevent physical disabilities in older adults. Public health policies should encourage older adults to engage in family affairs in addition to chronic disease control and maintenance of mental health.

## Figures and Tables

**Table 1:** Socio-demographic characteristics, health, and attitude toward aging in 2003 (*N* = 1635).

		*N* (%)
Age (mean ± SD)		65.32 ± 4.71
Gender		
	Male	847 (51.80)
	Female	788 (48.20)
Education		
	No Formal Education	445 (27.22)
	Elementary	933 (57.06)
	Middle/High School	150 (9.17)
	College Level or Higher	107 (6.54)
Marital Status		
	Single	328 (20.06)
	Married/Couple	1307 (79.94)
Work Status		
	Unemployed	1187 (72.60)
	Working	448 (27.40)
Income		
	None	1191 (72.84)
	Yes	441 (26.97)
	Refused to Answer	3 (0.18)
Living Alone Statement		117 (7.16)
Self-Described Health		
	Good	674 (41.22)
	General	552 (33.76)
	Poor	409 (25.02)
Number of Diseases		
	None	322 (19.69)
	More than One	1313 (80.31)
Pain		
	None	829 (50.70)
	Yes but Tolerable	784 (47.95)
	Yes and Intolerable	22 (1.35)
Hospitalization within the Past Year		
	No	1433 (87.65)
	Yes	202 (12.35)
Depression Status		
	None	73 (4.46)
	Low-Level Depression	1244 (76.09)
	High-Level Depression	318 (19.45)
Physical disability		
	Mobility limitations	1618 (61.99)
	ADL limitations	71 (2.72)
I agree with…		
	Children providing expenses	1456 (89.05)
	Keeping property for respect	1388 (84.89)
	Assisting with taking care of grandchildren	1445 (88.38)
	Avoiding living with married children	643 (39.33)
	Financial self-reliance	1247 (76.27)
	Young people today pay less respect to older people.	1222 (74.74)
	The government takes good care of the elderly.	954 (58.35)
	Living with children	1307 (79.94)
	Widower’s remarriage	357 (21.83)
	Widow’s remarriage	272 (16.64)

**Table 2:** Baseline attitudes toward aging in 2003 and covariates in predicting 2007 physical disability.

Variables	Mobility Limitation		ADL Limitation	
	aOR (95% CI)	*p*	aOR (95% CI)	*p*
Age	1.09 (1.06–1.12)	<0.0001	1.10 (1.05–1.17)	<0.01
Gender		<0.0001		0.15
Male	Reference		Reference	
Female	1.96 (1.49–2.57)		1.48 (0.87–2.52)	
Education				
No Formal Education	Reference		Reference	
Primary	0.81 (0.61–1.08)	0.15	0.80 (0.49–1.32)	0.40
Middle/High school	0.72 (0.45–1.14)	0.17	0.70 (0.22–2.18)	0.54
College or Higher	0.63 (0.36–1.09)	0.10	0.29 (0.03–2.36)	0.25
Married/Couple	1.26 (0.90–1.77)	0.17	1.04 (0.57–1.91)	0.88
Working	0.62 (0.31–1.22)	0.17	2.45 (0.53–11.2)	0.25
With Income				
None	Reference		Reference	
Yes	1.05 (0.53–2.07)	0.88	0.13 (0.02–0.69)	0.02
Refused to Answer	0.33 (0.02–4.47)	0.41	<0.0 (<0.0->999)	0.99
Living Alone	0.90 (0.54–1.50)	0.70	1.44 (0.62–3.33)	0.39
Self-Rated Health				
Good	Reference		Reference	
General	1.34 (1.02–1.76)	0.03	2.21 (1.07–4.55)	0.03
Poor	2.04 (1.45–2.88)	<0.0001	3.93 (1.85–8.33)	<0.01
More than one Disease	1.35 (1.00–1.83)	0.05	1.1 (0.48–2.51)	0.82
Pain				
None	Reference		Reference	
Yes but Tolerable	1.76 (1.37–2.27)	<0.0001	0.93 (0.53–1.64)	0.82
Yes and Intolerable	1.46 (0.51–4.20)	0.48	3.46 (0.97–12.3)	0.05
Hospitalization within the Past Year	1.22 (0.84–1.79)	0.29	1.34 (0.75–2.39)	0.32
Depression Status				
None	Reference		Reference	
Low-Level Depression	0.82 (0.47–1.43)	0.49	0.44 (0.16–1.19)	0.11
High-Level Depression	1.04 (0.56–1.95)	0.89	0.76 (0.27–2.12)	0.60
Physical Disability in 2003	---		---	
Mobility limitations	2.73 (2.12-3.52)	<0.0001	---	
ADL limitations	---		4.48 (1.79–11.1)	<0.01
Attitudes toward Aging in 2003 (Disagree reference to Agree)				
Children Providing Expenses	0.89 (0.60–1.30)	0.56	0.72 (0.28–1.86)	0.50
Keeping Property for Respect	0.91 (0.65–1.26)	0.58	0.80 (0.38–1.65)	0.55
Assisting with Taking Care of Grandchildren	1.28 (0.87–1.86)	0.20	2.46 (1.31–4.60)	<0.01
Avoid Living with Married Children	1.08 (0.84–1.39)	0.51	1.05 (0.63–1.73)	0.84
Financial Self-Reliance	0.67 (0.50–0.90)	0.01	0.99 (0.58–1.70)	1.00
Young People Today Pay Less Respect to Older People.	0.86 (0.66–1.13)	0.31	0.86 (0.49–1.50)	0.60
The Government Takes Good Care of The Elderly.	1.13 (0.89–1.44)	0.29	0.92 (0.56–1.51)	0.76
Living with Children	1.05 (0.77–1.43)	0.73	0.60 (0.29–1.24)	0.17
Widower’s Remarriage	1.43 (0.92–2.25)	0.11	1.15 (0.49–2.66)	0.74
Widow’s Remarriage	0.76 (0.46–1.26)	0.30	1.16 (0.42–3.19)	0.77

aOR: adjusted odds ratio.

## Data Availability

Restrictions apply to the availability of these data. Data was obtained from Taiwan Health Promotion Administration and are available at https://www.hpa.gov.tw/EngPages/Index.aspx with the permission of Taiwan Health Promotion Administration.
